# Therapeutic Efficacy and Biodistribution of Paclitaxel-Bound Amphiphilic Cyclodextrin Nanoparticles: Analyses in 3D Tumor Culture and Tumor-Bearing Animals In Vivo

**DOI:** 10.3390/nano11020515

**Published:** 2021-02-18

**Authors:** Gamze Varan, Cem Varan, Süleyman Can Öztürk, Juan M. Benito, Güneş Esendağlı, Erem Bilensoy

**Affiliations:** 1 Department of Pharmaceutical Technology, Faculty of Pharmacy, Hacettepe University, 06100 Ankara, Turkey; isikgamze@hacettepe.edu.tr (G.V.); varancem@hacettepe.edu.tr (C.V.); 2 Research and Application Center for Advanced Technologies (HUNITEK), Hacettepe University, 06100 Ankara, Turkey; scanozturk@hacettepe.edu.tr; 3 Institute for Chemical Research, Consejo Superior de Investigaciones Científicas (CSIC), University of Sevilla, Av. Américo Vespucio 49, 41092 Sevilla, Spain; juanmab@iiq.csic.es; 4 Cancer Institute, Department of Basic Oncology, Hacettepe University, 06100 Ankara, Turkey; gunese@hacettepe.edu.tr

**Keywords:** tumor targeting, cyclodextrin, nanoparticle, paclitaxel, biodistribution, breast tumor induced animal model

## Abstract

The uniqueness of paclitaxel’s antimitotic action mechanism has fueled research toward its application in more effective and safer cancer treatments. However, the low water solubility, recrystallization, and side effects hinder the clinical success of classic paclitaxel chemotherapy. The aim of this study was to evaluate the in vivo efficacy and biodistribution of paclitaxel encapsulated in injectable amphiphilic cyclodextrin nanoparticles of different surface charges. It was found that paclitaxel-loaded amphiphilic cyclodextrin nanoparticles showed an antitumoral effect earlier than the drug solution. Moreover, the blank nanoparticles reduced the tumor growth with a similar trend to the paclitaxel solution. At 24 h, the nanoparticles had not accumulated in the heart and lungs according to the biodistribution assessed by in vivo imaging. Therefore, our results indicated that the amphiphilic cyclodextrin nanoparticles are potentially devoid of cardiac toxicity, which limits the clinical use and commercialization of certain polymeric nanoparticles. In conclusion, the amphiphilic cyclodextrin nanoparticles with different surface charge increased the efficiency of paclitaxel in vitro and in vivo. Cyclodextrin nanoparticles could be a good candidate vehicle for intravenous paclitaxel delivery.

## 1. Introduction

Paclitaxel (PCX) is an effective anticancer drug used in many types of cancer including breast, ovarian, lung, head, and neck cancers. The unique PCX action mechanism is based on the abnormal microtubule stabilization that blocks mitotic progression leading to either the G0-phase of the cell cycle without cell division or, eventually, apoptosis [1]. The solubility of PCX in aqueous media is very low (<0.1 µg/mL) [2], which limits its injectability and bioavailability, so Cremophor EL^®^ and ethanol are used in commercial formulations as co-solvents to increase the solubility. Clinical side effects such as the hypersensitivity reactions, nephrotoxicity, and neurotoxicity [3] of PCX are also associated with co-solvents, even though these co-solvents are not sufficient to avoid the re-crystallization of PCX when diluted with saline or dextrose solution for intravenous infusion. The drug remaining recrystallized in the applied area causes insufficient doses to be carried to the tumor as well as causing necrosis and pain in the injection site [4]. Novel PCX formulations that are safer and more effective in lower doses are needed to overcome this vicious circle and increase treatment efficiency. To date, PCX is marketed in different nanoplatforms for parenteral delivery against various cancers: polymeric nanoparticles Abraxane^®^), polymeric micelles (Cynviloq^®^, Paclical^®^, Genexol^®^ and Nanoxel^®^), and liposomes (Lipusu^®^).

The tumors not only consist of malignant cells but also non-transformed cells such as fibroblasts, epithelial cells, endothelial cells, and pericytes. Therefore, a better targeting of this complex microenvironment, which is associated with tissue/cell interaction and penetration, is required for the improvement of anti-cancer therapeutics efficacy [5]. Even though in advanced cancers, the conventional chemotherapy is the first choice in clinics, the desired overall therapeutic success is not frequently achieved. Notably, systemic toxicity is observed due to the non-selectivity of anti-cancer drugs with a large volume of distribution and the side effects of the co-solvents. Additionally, chemoresistance is the most important obstacle to be overcome in order to provide successful chemotherapy. The nanoparticulate drug delivery systems such as Doxil (Doxorubicin-loaded PEGylated nano-liposome), Abraxane (nab-Paclitaxel), Marqibo (Vincristine sulfate liposome), DaunoXome (daunorubicin citrate liposome), and Onivyde (irinotecan liposome) have been developed and approved for increasing the efficacy of chemotherapeutics and for overcoming the drug resistance.

Nanoparticulate drug delivery systems have been a frequently studied approach for cancer treatment [6,7]. They can overcome the problems caused by conventional chemotherapy as a result of their unique physicochemical properties. Nanoparticles have various advantages such as small and adjustable particle sizes, modifiable surfaces, being prepared from a wide range of natural and synthetic polymers, high cellular penetration, and protecting the drug from physical and biological factors [8,9,10]. Cyclodextrins (CD), natural cyclooligosaccharides obtained from the enzymatic digestion of starch, have been profusely used in nanoparticle preparation for drug delivery purposes [11,12]. CDs feature rigid hollow-shaped troncoconic structures capable of hosting hydrophobic molecules in their nanometric cavity and stabilizing them in polar media. In addition, their dense hydroxyl display can be selectively functionalized to render an array of amphiphilic structures featuring spontaneous self-assembling capabilities in an aqueous environment, whose functional properties (i.e., stability, encapsulation capabilities, surface, interaction abilities, or environmental sensitivity) can be tailored. In fact, these nanodelivery systems were applied for the delivery of various anticancer drugs such as PCX [13,14,15], erlotinib [16], camptothecin [17,18,19], docetaxel [20], genistein [21], sorafenib [22], and quercetin/silibinin [23]. In addition, it has been preferred in studies to increase the effectiveness of various molecules besides anti-cancer drugs in different strategies such as gene silencing or phototherapy in cancer treatment [24,25,26,27,28,29,30].

The main purpose of the current study is to evaluate the efficacy and distribution of the PCX-encapsulated amphiphilic CD nanoparticles of different surface charges in 3D breast cancer cell cultures and breast tumor-bearing animals. Our results underline the superiority of the positively charged amphiphilic CD nanoparticles for delivering the PCX into the tumor site, avoiding its distribution amongst vital organs, and increasing its anti-cancer efficacy. For this purpose, two different amphiphilic βCDs were used in the study. Non-ionic 6OCaproβCD and cationic PC βCDC6 were used in our previous studies to create nano-sized carrier systems for anti-cancer drugs through oral or parenteral administration routes [13,15,16,17,19,31]. Our formulation development studies revealed both CDs as optimal carriers for PCX in our previous studies through cell culture studies and detailed in vitro characterization data including particle size, drug-loading efficiency, drug release profile, stability, hemolysis, and cytotoxicity in healthy cells [15,31]. Moreover, in our previous papers, it was determined that these two amphiphilic CD derivatives even in blank form had a high affinity to cholesterol microdomains in the cell membrane in human breast cancer cells (MCF-7), as well as removing cholesterol from the membrane and inducing apoptosis in cells in a dose-dependent manner that can be beneficial in fighting multidrug resistance based on cell membrane rigidity [32,33]. In this study, the antitumoral activities of anionic and cationic amphiphilic CD nanoparticles dispersions, which are ideal carrier systems for PCX, were evaluated for tumoral penetration in 3D spheroid tumor culture and antitumoral and antimetastatic efficacy in tumor-induced mice studies. In addition, the effect of different surface charge properties on the biodistribution and tumoral accumulation of injectable amphiphilic nanoparticles were also investigated and visualized in comparison to solution form.

## 2. Materials and Methods

Non-ionic amphiphilic CD, Heptakis(6-O-hexanoyl)cyclomaltoheptaose (6OCaproβCD) (Molecular Weight (MW): 1822 g/mol), and Heptakis[6-cysteaminyl-2,3-O-hexanoyl]cyclomaltoheptaose (PC βCDC6) (MW: 3178.15 g/mol) were synthetized as described previously in University of Sevilla, Spain [16,19,34]. Paclitaxel (>99% powder, MW: 853.91 g/mol) was purchased from LC Laboratories, Woburn, MA, USA. Matrigel^®^ Basement Membrane Matrix (356234) was obtained from Corning, NY, USA. Poly (2-hydroxyethyl methacrylate) (poly-HEMA) (P3932) was purchased from Sigma-Aldrich, St. Louis, MO, USA. Dulbecco’s modified Eagle medium (DMEM) (D5796, St. Louis, MO, USA), supplemented with 10% (*v*/*v*) fetal bovine serum (FBS) (F7524, Sigma-Aldrich, St. Louis, MO, USA) and 1% penicillin /streptomycin (P4333, Sigma-Aldrich, St. Louis, MO, USA) was used for all cell culture studies (hereinafter referred to as “complete DMEM”). Roswell Park Memorial Institute Media (RPMI-1640) (R8758) was obtained from Sigma-Aldrich, St. Louis, MO, USA. All other chemicals used were of analytical grade and obtained from Sigma-Aldrich, St. Louis, MO, USA. Ultrapure water was used as obtained from Millipore Simplicity 185 Ultrapure Water System (Millipore, Molsheim, France).

### 2.1. Cell Culture Studies

#### 2.1.1. Determination of PCX IC_50_ Value on 4T1 Cell Line

In order to determine the half maximal inhibitory concentration (IC_50_) value of PCX solution, 4T1 cells were seeded in a 96-well cell culture plate (1 × 10^4^ cells/well) in complete DMEM (100μL) and were allowed to attach overnight. Then, the medium was replaced with 7 different dilutions of PCX stock solution (4mg/mL in dimethyl sulfoxide (DMSO)) in complete DMEM (in a range from 3.9 to 250 µM). In addition, cells were incubated with complete DMEM containing the same concentration of DMSO, and the results were normalized with DMSO groups. After 48 h of incubation time, cell viability was determined with WST-1 assay. For this purpose, 10 μL WST-1 reagent was added in each well, and cell viability was determined with a microplate reader at 450 nm. Cells treated with complete DMEM were considered as control and 100% viable. The IC_50_ value of PCX solution was calculated by using GraphPad Prism version 7. (GraphPad Software Inc., San Diego, CA, USA).

#### 2.1.2. Anticancer Activity of Amphiphilic Cyclodextrin Nanoparticles

The antitumoral activities of PCX loaded amphiphilic CD nanoparticles were determined on the 4T1 (ATCC^®^ CRL-2539™, Manassas, VA, USA) mouse mammary tumor cell line, which was further used to induce a tumor model in mice. For this purpose, cells were grown in complete DMEM. Then, cells were seeded at a density of 5 × 10^3^ cells/well in complete DMEM (100 µL) into each well of 96-well plates. Then, the cells were incubated for 24 h in a 5% CO_2_ incubator at 37 °C. At the end of 24 h, the cell culture medium in the wells was replaced with fresh medium containing PCX alone or PCX-loaded amphiphilic CD nanoparticles. After 48 h incubation, WST-1 (10 μL) was added into the cells. The cells were incubated for about 2 h, and then, the absorbance at 450 nm was determined with a microplate reader, and cell viability was calculated. The cells incubated with complete DMEM were evaluated as the control, and the viability of these groups was accepted as 100%.

The effect of PCX alone or PCX-loaded amphiphilic CD nanoparticles on 4T1 cells was also monitored microscopically with a Viability/Cytotoxicity Assay Kit (30002, Biotium, Fremont, CA, USA). In order to detect live and dead cells in 4T1 cells, cells were incubated with nanoparticle formulations for 48 h; then, the medium was removed, and 200 µL of dye mixture was added to each well and incubated for 45 additional minutes. After the incubation, groups of cells were visualized by fluorescence microscopy.

#### 2.1.3. Migration Assay

The inhibitory effect of amphiphilic CD nanoparticles on the migration and invasion ability of 4T1 cells was measured by a wound healing-based method [35]. For this purpose, 4T1 cells were plated in 6-well culture plates at a density of 1 × 10^6^ cells per well and cultured overnight to form a confluent monolayer. The following day, a vertical scratch was generated across the center of each well using a 200 µL pipette tip to form a cell-free zone and then washed with phosphate-buffered saline (PBS) twice to remove non-adherent exfoliated 4T1 cells. Cells were incubated with the fresh medium containing drug-loaded nanoparticle formulations for 48 h. The plates were imaged using a microscope, and migration was quantified as the percent decrease in the mean migration zone area.

#### 2.1.4. 3D Tumor Culture Studies

The antitumoral activity and tumoral penetration ability of PCX-loaded amphiphilic CD nanoparticles and PCX solution were determined against a scaffold-based 3D in vitro tumor model using MCF-7 human breast cancer cells by our group previously [15]. In this study, 3D tumor culture was formed with 4T1 cells that were used in the animal model. Briefly, poly (2-hydroxyethyl methacrylate) coated U-bottom cell culture plates were prepared. Then, 1 × 10^4^ cells/200 µL medium per well was added into the each well containing Matrigel^®^ Basement Membrane Matrix equal to 3% of the total medium. Subsequently, the cells were spun at 1000 rpm for 10 min using a plate centrifuge. After centrifugation, the cells were removed to the incubator, and the media were replaced with fresh for 7 days for growth. At the end of the incubation time, the medium was removed from the spherical tumors, and the medium containing the nanoparticle formulation was added. After 48 h, cell viability in tumors was determined by the WST-1 method and living/dead cells in tumors were imaged by confocal microscopy (Zeiss LSM 5, Pascal, Oberkochen, Germany) after staining, as described above.

### 2.2. In Vivo Studies

#### 2.2.1. Antitumoral Activities of Amphiphilic Cyclodextrin Nanoparticles

Female BALB/c mice (22–24 g, 6–8 weeks of age) (Kobay AŞ, Ankara, Turkey) were maintained in cages at 22 ± 3 °C, 55% relative humidity of under a 12-h dark/light cycle. Mice were allowed free access to food and water. All the experiments and handling of animals were performed following the approval by Hacettepe University Local Ethical Committee (approval number: 2019/02-10).

4T1-Red-FLuc Bioware^®^ Brite Cell Line (Perkin-Elmer) were used for in vivo studies. Luciferase-expressing cells were grown in an incubator containing 5% CO_2_ at a constant temperature of 37 °C in medium (completed RPMI with 10% FBS and 1% penicillin/streptomycin). A syngeneic tumor model was developed in 6–8-week-old female BALB/c mice to test the antitumor effect of amphiphilic CD nanoparticles. After subcutaneous injection of 3 × 10^5^ 4T1-Red-FLuc cells in 100 µL PBS to the inguinal fat pad of mice, tumor development was followed until reaching 0.5 cm diameter before nanoparticle injection.

In order to test the antitumoral effect, mice were separated into seven groups including; blank 6OCaproβCD nanoparticles (n = 7); blank PC βCDC6 nanoparticles (n = 7); PCX-loaded 6OCaproβCD nanoparticles (n = 8); PCX-loaded PC βCDC6 nanoparticles (n = 8); PCX solution in Cremophor^®^EL: EtOH (1:1 *v*/*v*) (n = 8); Cremophor^®^EL:EtOH (1:1 *v*/*v*) (n = 8) and untreated control group (n = 7) randomly. All injections were performed three times a week intraperitoneally (1.25 mg/kg/day). Mice were weighed, and tumor growth was measured by using a caliper twice a week. In addition, tumor growth was monitored with D-luciferin (150 mg/kg, Biovision, Milpitas, CA, USA) injection intraperitoneally during the experiment. Mice were anesthetized, and luciferase activities were recorded three times a week using an in vivo imaging system (Newton 7.0, Vilber, Collégien, France).

#### 2.2.2. Biodistribution of Amphiphilic Cyclodextrin Nanoparticles

The biodistribution of amphiphilic cyclodextrin nanoparticles was performed by the injection of Flamma^®^ 774 NHS ester dye (PWS1603, BioActs, Incheon, Korea)-loaded 6OCaproβCD and PC βCDC6 nanoparticles intravenously through the tail vein. The dye solution was used as a control group. Mice were monitored at 2, 6, and 24 h after injection using an in vivo imaging system (Newton 7.0) under anesthesia (n = 3). To capture 4T1-Red-FLuc cells’ bioluminescence in live mice at 24 h, D-Luciferin was injected intraperitoneally, and tumor–dye interaction was compared. Mice were sacrificed, and organs were dissected at the 6th and 24th hours. Photographs of organs were taken under near-infrared filter, and a mean florescence intensity of Flamma^®^ 774 NHS ester dye was calculated using the ImageJ Fiji program (Madison, WI, USA).

### 2.3. Statistical Analysis

For both cell culture assay and in vivo studies, a Student’s *t* test was used for multiple comparisons using GraphPad Prism 7 (GraphPad Software Inc., San Diego, CA, USA). A value of *p* < 0.05 was considered statistically significant. Results were expressed as mean ± SD (standard division)

## 3. Results and Discussion

### 3.1. Physicochemical Properties of the Nanoparticles

The formulation, in vitro safety, and anti-cancer activity of the amphiphilic CD nanoparticles used in this paper were determined and optimized in our previous studies [16,31,33]. Blank and drug-loaded amphiphilic CD nanoparticles were prepared using the nanoprecipitation method, as described previously [31]. Monodisperse PCX-loaded amphiphilic CD nanoparticles were reproducibly produced from 6OCaproβCD (average hydrodynamic diameter 113 ± 4 nm) and PC βCDC6 (82 ± 2 nm), respectively. In addition, the zeta potential (ζ) of nanoparticles was determined as −29 ± 2 mV for 6OCaproβCD and +62 ± 1 mV for PC βCDC6 as a result of the analysis by dynamic light scattering (DLS), using a Malvern ZetaSizer Nano ZS (Malvern Instruments, Malvern, UK). The efficiency of paclitaxel loading (> 40%) and stability of the nanoparticles (over 30 days) were also shown (Table 1) [31,33].

### 3.2. Cell Culture Studies

#### 3.2.1. Determination IC_50_ Value of Paclitaxel on 4T1 Cell Line

**Figure 1:**
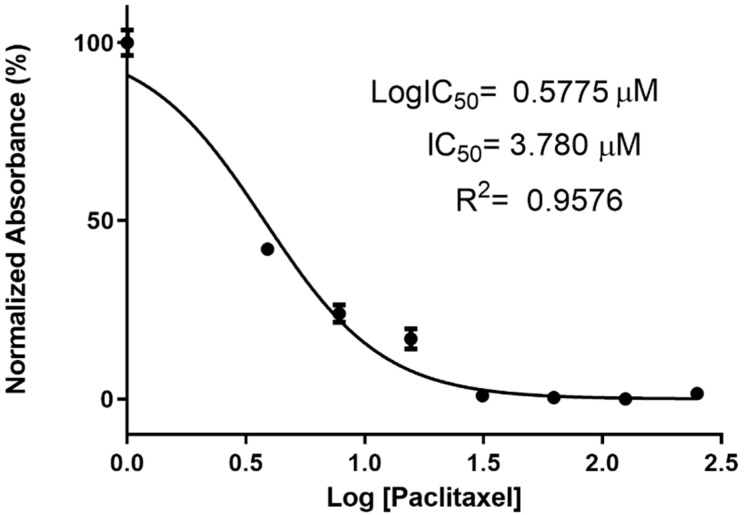
The half maximal inhibitory concentration (IC_50_) value of paclitaxel solution on 4T1 murine breast cancer cell line at 48 h. Cells were treated with the different concentrations of Paclitaxel (250, 125, 62,5, 31.25, 15.6, 7.8, and 3.9 µM). The absorbance was determined as a result (n = 6, mean ± SD).

To optimize the concentration of PCX-loaded CD nanoparticles to be used in further experiments, the IC_50_ value of PCX was calculated on the 4T1 breast cancer cell line with serial dilution of PCX solution in DMSO. Non-treated cells and cells incubated with DMSO-containing media were used as control groups. Normalized absorbance against PCX concentration was plotted, and the IC_50_ value of PCX was calculated from the logarithmic trendline of the graphs by using GraphPad Prism 7. Viability in the control group was taken as 100% cell proliferation. According to the results shown in Figure 1, the IC_50_ value of PCX was determined as 3.78 µM for 4T1 cells. Thus, for the rest of the cell culture assays, nanoparticles were diluted with complete DMEM to contain 3.78 µM PCX to ensure application in the range of IC_50_.

#### 3.2.2. Anti-Cancer Activity of Amphiphilic Cyclodextrin Nanoparticles

**Figure 2:**
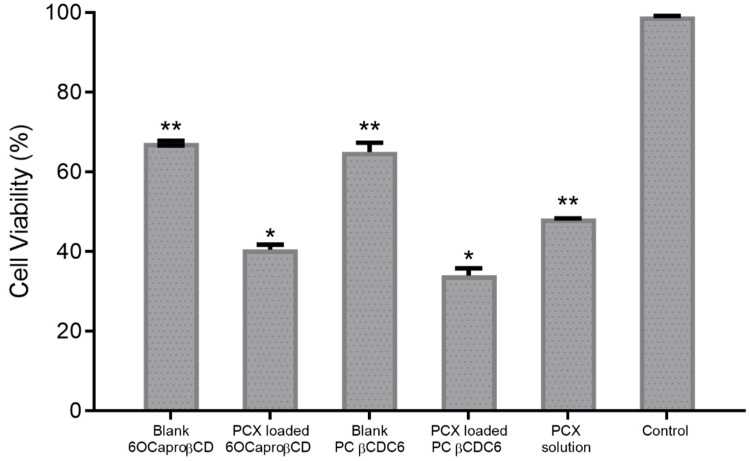
Anticancer effect of blank or paclitaxel-loaded amphiphilic cyclodextrin nanoparticles and paclitaxel solution against 2D 4T1 murine breast cancer cell line. Cell viability was evaluated by WST-1 assay. (n = 6, mean ± SD), (* *p* < 0.05 compared with PCX solution and ** *p* < 0.05 compared with control group).

The viability of cells treated with PCX-loaded anionic and cationic nanoparticles at the same PCX effective concentration (3.78 μM) was 40% ± 1.2% and 34% ± 2.8%, respectively (Figure 2). These findings show that the anticancer activity of PCX was enhanced by encapsulation into amphiphilic CD nanoparticles of different charge. Moreover, cell viability was reduced to 67% ± 0.6% and 65% ± 2.3% in the groups treated with blank anionic and blank polycationic nanoparticles, respectively. Results indicate that both of the CD nanoparticles caused higher cancer cell mortality than free PCX solution indicated by the significant difference between the PCX-loaded amphiphilic CD nanoparticles and free PCX (*p* < 0.05).

When the obtained data were evaluated, statistical difference was found between the two PCX-loaded CD nanoparticle formulations with the polycationic CD causing higher cell death. On the other hand, the effect of blank CD nanoparticle formulations on cell viability was similar to each other (*p* > 0.05). Thus, it is thought that viability in cells incubated with drug-loaded nanoparticles is dependent on the amount of PCX uptake by cells rather than the anti-cancer activity of the CDs itself. This uptake is inversely correlated with the size of the nanoparticles. Both nanoparticle formulations contain equal amounts of PCX (3.8 µM), and therefore, cell viability was reduced depending on the uptake of PCX. It is known that zeta potential plays an important role in the interaction with biological membranes, cellular uptake, and opsonization. The net surface charge of nanoparticles directly affects cellular uptake properties. The cationic surface charge improves the cellular penetration of nanoparticles by increasing the electrostatic interaction with the plasma membrane, which has a negative surface charge due to its double-layer phospholipid chains [36,37]. However, when the structures of CD derivatives and anti-cancer activities are considered together, it is thought that other factors may influence cell viability. In a previous study, we have shown that the positively charged PCX-loaded CD nanoparticles exhibited a more pronounced anti-cancer activity in 2D and 3D MCF-7 cells [15,31]. More recently, we have also demonstrated that positively charged nanoparticles were more effective against 2D and 3D liver and lung cells cultures and caused a significant decrease in IC50 value of erlotinib [16]. Taken together, our findings suggest that positively charged nanoparticles enhance anti-cancer drug uptake and decrease cell proliferation. Studies showed that CD nanoparticles with positive surface charge also show success in oral chemotherapy. Camptothecin-loaded amphiphilic CD nanoparticles were developed and evaluated for oral chemotherapy in breast and colorectal carcinoma treatment. The results suggest that the drug loading and cellular interaction of the CD nanoparticles can easily be modulated by coating with a positively charged biocompatible material with penetration enhancer properties such as chitosan. Thus, camptothecin-loaded CD nanoparticles can be effective in improving the oral bioavailability and decreasing the dosing frequency, thereby minimizing the dose-dependent adverse effects and maximizing the patients’ compliance [17,18,19].

**Figure 3:**
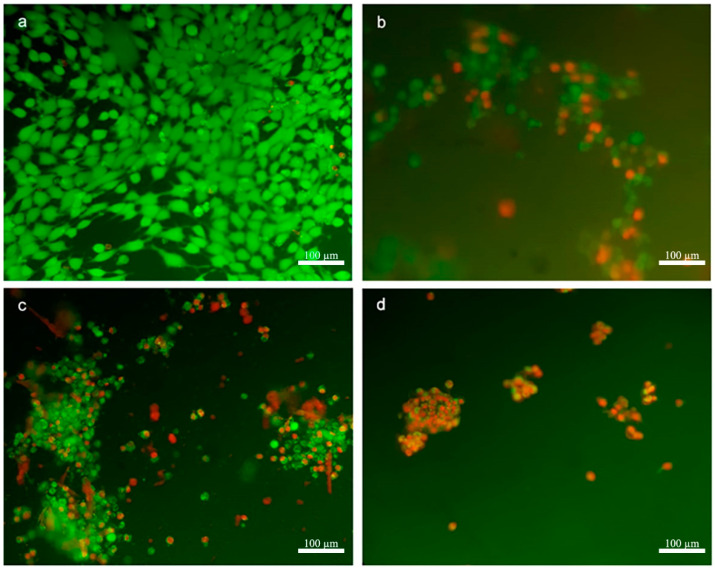
Live/dead analysis of 4T1 cells using double staining of Calcein AM and Ethidium Homodimer-1 (EthD-1) after treatment with different formulations: control group treated with only complete Dulbecco’s modified Eagle medium (DMEM) (**a**), paclitaxel solution-treated cells (**b**), paclitaxel-loaded negatively charged cyclodextrin nanoparticles treated group (**c**), and paclitaxel-loaded positively charged cyclodextrin nanoparticles treated group (**d**). Live cells stained with Calcein AM show green fluorescence and dead cells stained with EthD-1 show red.

The 4T1 cells were visualized by fluorescent microscopy to observe live/dead cells in groups treated with PCX-loaded nanoparticles or PCX alone. According to the results shown in Figure 3, the characteristic morphology of living 4T1 cells (green) was observed in the control group incubated only with complete DMEM. In other groups, which were treated with different amphiphilic CD formulations, dead cells stained with red color were observed along with living cells. It was observed that PCX-loaded CD nanoparticles have a negative influence on the morphological structures and colonization of 4T1 cells and cause a decrease in cell viability.

#### 3.2.3. Migration Assay

**Figure 4:**
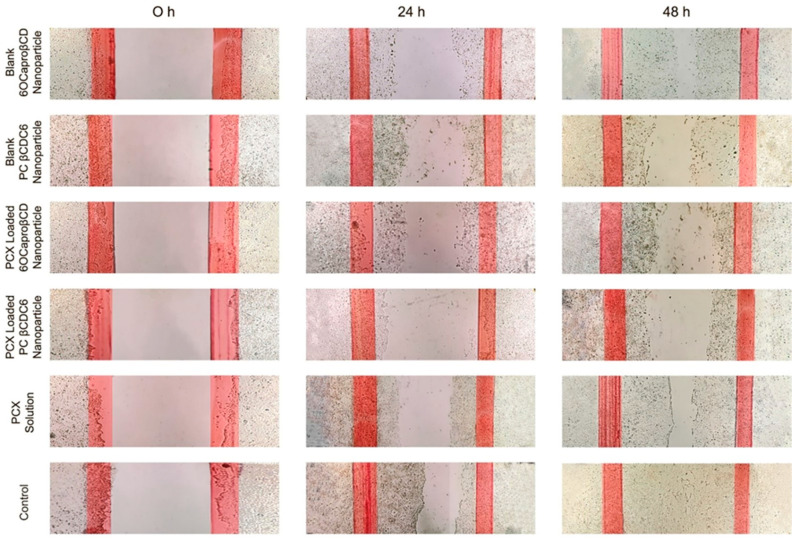
Suppression of migration and invasion of 4T1 murine breast cancer cells by blank or paclitaxel-loaded amphiphilic cyclodextrin nanoparticles in vitro. Wound-healing assay of 4T1 cells before and after blank nanoparticles, drug-loaded nanoparticles, paclitaxel solution, or complete DMEM treatment of up to 48 h.

The wound healing and migration experiment offer the opportunity to observe cell migration, which plays an important role in several processes in tumor development, such as neoangiogenesis and metastasis. In cell culture as well as in vivo, microtubules are required for cell migration [38]. In this regard, it is important to determine the efficacy of microtubule-stabilizing agents such as PCX in wound-healing assays. The effect of the developed PCX-loaded CD nanoparticles on the migration of 4T1 murine breast cancer cells was determined by wound-healing assay. The results obtained are shown in Figure 4. According to the microscopic images obtained for 48 h, it can be suggested that CD nanoparticles suppress the migration of 4T1 cells. Moreover, blank CD nanoparticles were also observed to inhibit cell migration and thus wound healing. At the end of 48 h, it was observed that the cell-free scraped area at the beginning of the experiment was completely covered with 4T1 cells in the control group incubated with complete DMEM only. On the contrary, blank or PCX-loaded CD nanoparticles inhibited the vertical migration capacity of 4T1 cells. It was observed that even blank CD nanoparticles negatively affected the migration of cells after 48 h. It was observed in studies performed with both blank and drug-loaded nanoparticles that the polycationic CD is more effective than the non-ionic CD. All formulations were more effective than the free PCX solution in suppressing the migration capacity of 4T1 cells.

#### 3.2.4. 3D Spheroid Culture

In vitro 3D spherical tumors can mimic the tumor microenvironment and natural tumor morphology, and they are developed to fill the gaps between 2D conventional cell culture and in vivo animal studies. Tumor growth and metastasis is a biological process controlled by the extracellular matrix (ECM), cancer cell, and stroma. In this biological process, the development and metastasis of cancer cells depends on many factors such as growth factors, hormones, and other cells within the ECM. By using this in vitro 3D spherical tumor model, cell–cell and cell–ECM interaction can be provided, and hierarchical arrangement can be simulated [39,40,41]. Cell-based studies are the main tool for evaluating the potential efficacy of a new drug compound. However, in order to obtain the most reliable results in cell culture studies, the analysis method should mimic the biological environment as well as possible. For this reason, 3D spherical tumor models are used in cancer studies where 2D conventional cell culture studies are insufficient. In this study, 3D in vitro cell culture studies were carried out before the in vivo model.

**Figure 5:**
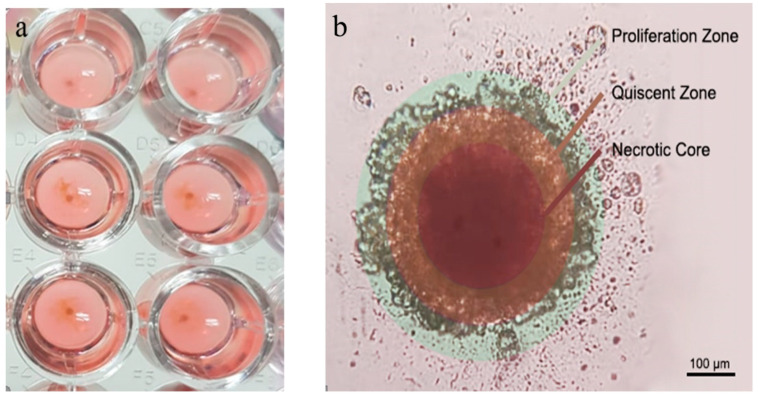
Individual 4T1 spheroids are formed in each well of microplates (**a**) also shown with different regions for proliferating, quiescent and necrotic zone (**b**). Murine breast cancer spheroid was formed by scaffold-based method, and the morphological structure was observed under the light microscope. Scale bar: 100 µM.

4T1 spherical tumors were prepared by the scaffold-based method in this study [15]. 4T1 spherical tumors formed as one nodule in each well of cell culture plate. Microscopic examination of nodules showed a spherical morphology and different zones: proliferation zone, quiescent zone, and necrotic core (Figure 5b).

**Figure 6:**
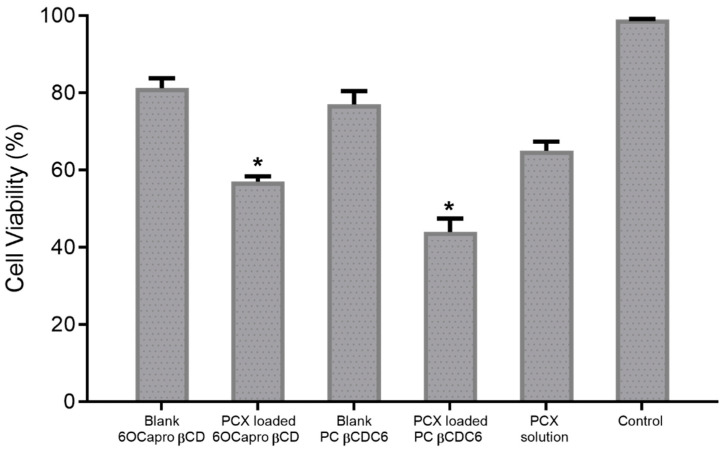
Anticancer effect of paclitaxel (PCX) solution and PCX-loaded amphiphilic cyclodextrin (CD) nanoparticles against 3D 4T1 spheroids for 48 h. Cells incubated with the complete DMEM were considered as control and 100% viable. Cell viability was determined with WST-1 colorimetric assay (n = 4; mean ± SD). * *p* < 0.05 compared with PCX solution.

4T1 spheroids were allowed to grow for 7 days and then incubated with the appropriate nanoparticle formulations or PCX solution for 48 h. Cell viability data obtained as a result of WST-1 analysis are shown in Figure 6. According to the results, cell viability was calculated as 57% ± 1.4%, 44% ± 3.5% and 65% ± 2.4% for PCX-loaded anionic, cationic CD nanoparticles, and PCX solution, respectively. In addition, the percentage of viable cells was found to be over 77% in groups treated with blank CD nanoparticles. In this context, it can be said that both nanoparticle formulations successfully deliver the PCX through the 3D and multilayer spherical tumor model and significantly reduce the cell viability in the tumor compared to the drug solution.

When the results of 2D and 3D homospheroid studies performed on 4T1 cells are compared, it is noticed that the difference is significant in terms of cell viability, and this is an expected result. The percentage of viability in cells incubated with PCX-loaded anionic CD nanoparticles increased from 40% in the 2D model to 57% in the 3D model. Similarly, this value increased from 34% to 44% for PCX-loaded cationic CD nanoparticles. In addition, similar results are also found with the PCX solution; in the 2D culture model, cell viability was determined as 49%, but in 3D tumor studies, the viability was determined as 65%. It is known that cells in 3D tumors typically have lower sensitivity to cytotoxic drugs than 2D monolayer cells. It has been argued that this difference has several causes, including reduced drug penetration, the development of hypoxic nuclei, and reduced growth [39]. It is also known that increased intercellular signaling by enhancing the cell–cell interaction of 3D cell culture is an important factor in decreased drug sensitivity in spherical tumors [42]. In this field, Green et al. incubated HT29 human colorectal adenocarcinoma cells with E-cadherin inhibitory antibody, and then, their sensitivity to various anticancer drugs was determined. It was shown that 3D colon cancer tumors have increased sensitivity to 5-fluorouracil, PCX, vinblastine, and etoposide as a result of the inhibition of E-cadherins, which is one of the adhesion molecules that provide intercellular connectivity [43].

While preparing 3D spherical tumors, Matrigel^®^ was used to provide ECM in the environment. Matrigel is derived from the basement membrane of Engelbreth–Holm–Swarm (EHS) mouse sarcoma cells and contains 60% laminin, 30% collagen IV, and 8% entactin. Collagen and laminin are essential components for cell growth and attachment. Entactin, on the other hand, is a bridging molecule that interacts with laminin and collagen IV and contributes to the structural arrangement of extracellular matrix molecules. Moreover, Matrigel^®^ contains perlecan, epidermal growth factor, insulin-like growth factor, fibroblast growth factor, tissue plasminogen activator, and other growth factors that are naturally present in the tumor structure. In this study, it was shown that the response of spherical tumors prepared in the presence of a scaffold such as Matrigel to the nanoparticulate drug delivery system is significantly different from the response of single-layered cells in conventional 2D cell culture and a more realistic indicative of the expected in vivo behavior of the nanoparticles.

**Figure 7:**
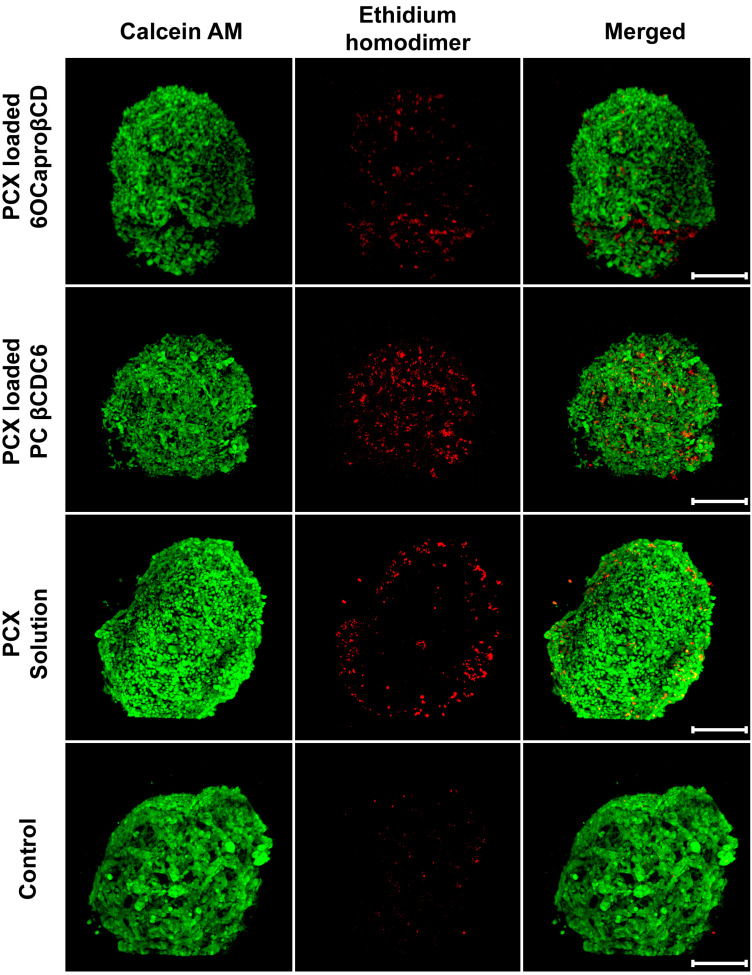
Antitumoral effectiveness of paclitaxel-loaded cyclodextrin nanoparticles and paclitaxel solution on 3D spherical breast tumor viability. Three-dimensional (3D) spheroids were incubated with complete DMEM alone observed as a control group. 4T1 cells were seeded at 1 × 10^4^ cells per well into 96-well U-bottom plates, formulations were added to the spheroids, and the plates were returned to the incubator for 48 h of culture. Calcein AM (live, green fluorescence) and ethidium homodimer (dead, red fluorescence) reagents were added to the wells, and plates were incubated for an additional 45 min (scale bar: 500 µM).

The antitumoral efficacy of PCX-loaded amphiphilic CD nanoparticles against a 3D spherical tumor model was also observed microscopically. The cells in the control group were incubated with medium alone. Living cells stained in green in the control group were observed in spherical tumors. As seen in Figure 7, it was observed that the dead cells were mostly in the proliferation zone of tumors treated with free PCX solution. Results in the group treated with PCX-loaded anionic CD nanoparticles are similar to the PCX solution. However, in the image of the PCX-loaded cationic CD nanoparticles shown in Figure 7, it is seen that the dead cells are in the inner parts of the tumor along with the surface. These images indicate that CD nanoparticles with positive surface charge can advance through the multilayered tumor layer and penetrate through the tumor layer.

According to the microscopic images, it can be said that in tumor spheroids treated with anionic CD nanoparticles, dead cells were mostly located on the tumor surface, but in tumors treated with cationic CD nanoparticles, dead cells were also located deep into the 3D tumor structure as well as near the surface. It is an expected result that the surface charge of nanoparticles increases tumor penetration. Positively charged nanoparticles interact with negatively charged components such as sialic acid, cholesterol, and phospholipids in the membrane structure of cells stronger than anionic nanoparticles [44]. Therefore, surface charge is very important in cellular and tumoral uptake and localization in subcellular units [45,46]. In our previous in vitro 3D tumor studies using human lung (A549), liver (HepG2), and breast (MCF-7) cancer cells, the efficacy of anticancer drug-loaded amphiphilic CD nanoparticles was determined [15,16]. In these studies, it was observed that CD nanoparticles have different antitumoral activities in cells depending on their surface charge, on the cancer cell type, and the drug they carry. Moreover, hydrophobic Nile red-loaded nanoparticles were prepared, and their penetration into a multilayer MCF-7-based spherical tumor was observed with confocal microscopy. It was observed that nanoparticles with a positive surface charge can penetrate the tumor deeply, which may be related to the results of this study, while it was determined that nanoparticles with negative surface charges were mostly uptaken by cells on the surface of the tumors. Therefore, dead cells (colored red) were observed at the tumor surface in the group treated with anionic CD but also at deeper levels in the group incubated with cationic CD nanoparticles.

### 3.3. In Vivo Studies

#### 3.3.1. Antitumor Efficacy of Paclitaxel-Loaded Amphiphilic Cyclodextrin Nanoparticles

**Figure 8:**
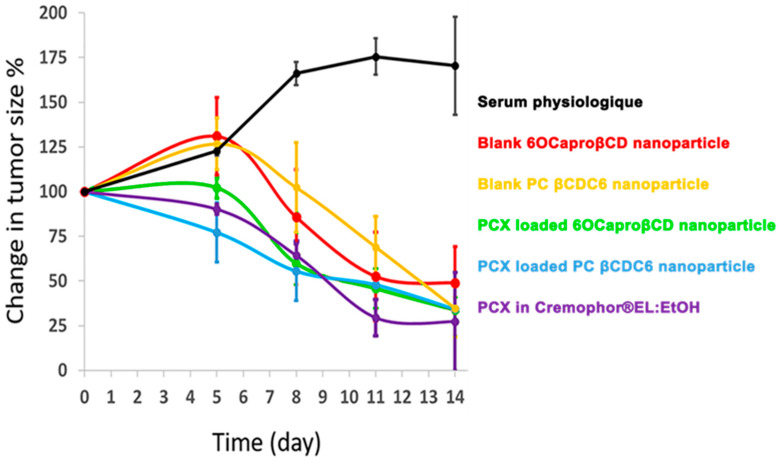
Change in the tumor size upon treatment with blank, paclitaxel-loaded cyclodextrin nanoparticles, and paclitaxel solution in Cremophor^®^EL:EtOH. Data were represented as mean ± SD.

**Figure 9:**
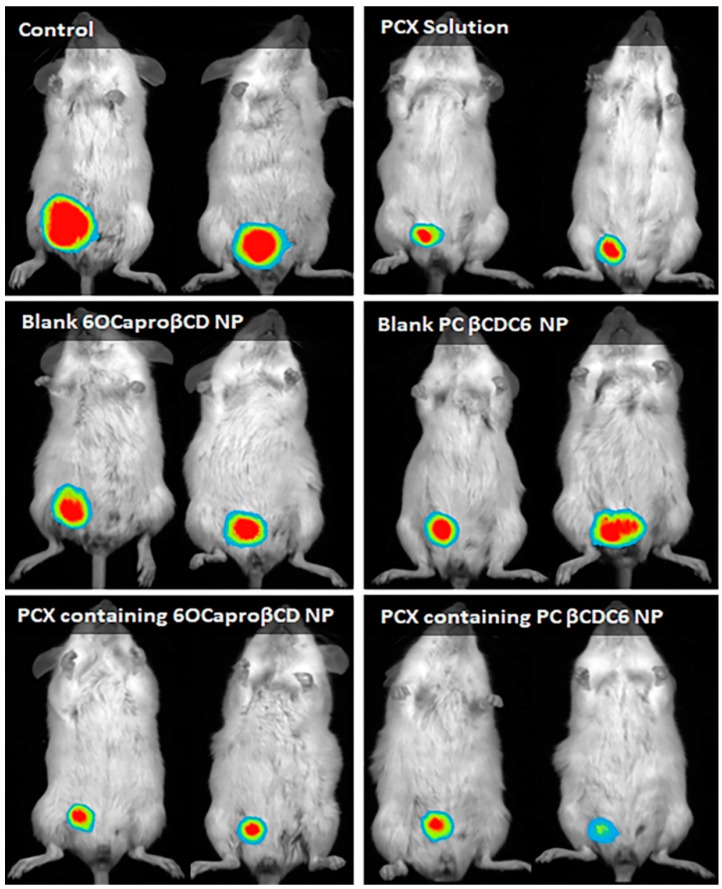
Visualization of the tumor mass on day 14 of the in vivo studies. The 4T1-Red-FLuc tumors were monitored with D-luciferin (150 mg/kg, Biovision) injection intraperitoneally (left colon day 0, right column day 8). Mice were anesthetized, and luciferase activities were recorded in an in vivo imaging system.

The blank and the PCX-loaded CD nanoparticles were administered to the tumor-bearing mice, and the change in tumor size was followed for 14 days (Figure 8 and Figure 9). On day 5, compared to the control groups that received physiological saline or blank CD nanoparticles, an approximately 25% reduction was observed in the tumor size of the mice treated with PCX-loaded nanoparticle or PCX solution (Figure 8). The most significant difference between the groups was achieved on day 8 in which the tumors continued to grow in the physiological saline control group; on the other hand, either the blank or the PCX-loaded CD nanoparticles reduced the tumor burden. In general, the PCX-loaded positively charged nanoparticles were the most efficient antitumor formulation, albeit not reaching the level of statistical significance (Figure 8). On day 14, the tumor size was reduced by 50% in all groups that were treated with blank or PCX-loaded CD formulations, or PCX solution. Collectively, the antitumor effect of the PCX-loaded amphiphilic CD nanoparticles was observed earlier than the PCX solution. Interestingly, in the long run, the blank CD nanoparticles were also capable of hindering the tumor growth (Figure 8 and Figure 9). Accordingly, Erdogar et al. showed that folate-targeted CD nanoparticles were better tolerated by animals and localized in the tumor area than PCX solution in Cremophor^®^EL [14]. These results support that the CD nanoparticles can be a good candidate for increasing the efficacy and safety of PCX therapy in breast cancer.

In our previous studies, it was observed that blank amphiphilic CD derivatives cause a selective antiproliferative effect in various cancer cells (MCF-7, HepG2, MDA-MB-231), although they do not have any toxic effects on healthy fibroblasts (L929) and healthy bladder cells (G/G1) [33]. With advanced cell culture studies performed with proteomic and metabolomic approaches, it was determined that this effect was caused by the cholesterol affinity of CDs [32]. It is known that amount of cholesterol in the membranes of cancer cells is higher than that in healthy cells. Moreover, the cholesterol content in the membrane also varies according to the drug resistance of the cancer. Todor et al. reported that the amount of cholesterol increased by 60% in resistant MCF-7 cells [47]. CDs are also used to manipulate the cholesterol composition in different cells in the literature.

In our previous study, the effects of blank 6OCaproβCD nanoparticles on MCF-7 cells were investigated by biochemical and proteomic tests. According to the results of the proteomics studies, it was determined that the protein levels of Heterogeneous nuclear ribonucleoproteins (hnRNP) and chromobox protein (CBX1) associated with apoptosis were increased, and hepatoma-derived growth factor (HDGF) was not affected. In addition, the findings obtained by RT-PCR showed that the 6OCaproβCD nanoparticle, which can be used as a nanoparticular drug carrier, does not trigger multidrug resistance. The effect of the metabolomic pathways affected by blank 6OCaproβCD nanoparticles on breast cancer cells was elucidated with Human Metabolome Database (HMDB) as serine biosynthesis, transmembrane transport of small molecules, metabolism of steroid hormones, estrogen biosynthesis, and phospholipid biosynthesis [32]. 

The in vivo antitumoral activity exhibited by the blank CD nanoparticles observed in this study confirms and strengthens the previous findings. PCX used as an anti-cancer model drug in this study is an effective chemotherapeutic. However, as emphasized in the literature, there are serious toxicity problems in its clinical application [3,4]. In the light of the findings obtained in this study, it can be said that even blank nanoparticles as well as PCX-loaded CD nanoparticles can be used as an alternative approach to PCX solution. In this way, it is believed that the toxicity caused by Cremophor^®^EL can be prevented.

#### 3.3.2. Biodistribution of Amphiphilic Cyclodextrin Nanoparticles

**Figure 10:**
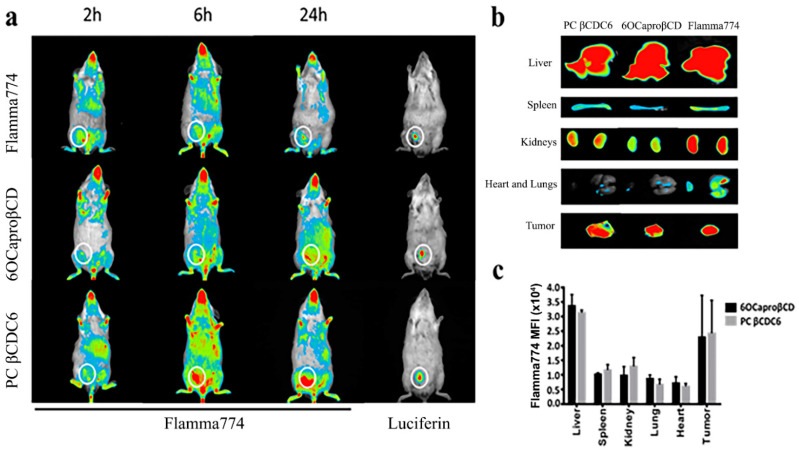
Biodistribution of amphiphilic cyclodextrin nanoparticles and dye solution in breast tumor-bearing mice for 24 h (**a**), accumulation of nanoparticles and dye solution in organs in mice that were sacrificed at the end of 24 h (**b**), and mean fluorescence intensity of Flamma^®^ 774 in organs (mean ± SD) (**c**). Tumor developing in mice highlighted in a white ring.

The biodistribution of the amphiphilic CD nanoparticles, 6OCaproβCD and PC βCDC6, was determined following loading with Flamma^®^ 774 dye. Even though there was only a slight difference at the early time point (2 h) between the dye solution and the CD nanoparticles, at the later time point, both amphiphilic CD derivatives were found to more competently accumulate in the tumor area (Figure 10).

Major organs were examined after the mice were sacrificed, and it was observed that the dye solution accumulated in the heart and lungs, but on the contrary, the nanoparticles were barely detected in the lungs and heart (Figure 10b,c). This is an unexpected result when considering the surface charges of cationic nanoparticles. It is known that nanoparticles with a cationic surface charge accumulate in the lungs due to the surfactant [48]. When the kidneys were examined, it was observed that the highest accumulation was in the mice administered with the dye solution. Renal involvement was lesser with negatively charged nanoparticles than cationic nanoparticles. This may be the result of both the surface charge and the small particle size of the cationic CD derivative. As emphasized previously, amphiphilic CD nanoparticles had mean diameters of 113 ± 4 nm and 82 ± 2 nm for 6OCaproβCD and PC βCDC6, respectively. In addition, zeta potential (ζ) of nanoparticles were determined as −29 ± 2 mV for 6OCaproβCD and +62 ± 1 mV for PC βCDC6 [31]. The organ distribution of the nanoparticles suggests low cardiotoxicity and lung accumulation after a single injection and 24 h monitoring, which can be favorable for a systemic nanomedicine since some polymeric nanoparticles are associated with cardiac toxicity, limiting their clinical use and commercialization.

## 4. Conclusions

In this study, nano-sized carrier systems for PCX were prepared using amphiphilic CD derivatives with different surface charges, and their efficacy was determined by in vitro and in vivo studies. The antitumoral activities of two different CD derivatives, which were determined by our group to be safe carriers for cancer treatment in previous studies, were supported by in vivo studies. In the light of the findings obtained, it can be said that both CD derivatives are more effective than PCX solution in a 3D spherical tumor model conducted with breast cancer cells. Moreover, in vivo studies, we observed that both blank and anti-cancer drug-loaded nanoparticles significantly reduce the tumor volume. These findings support that both CD derivatives can provide effective cancer treatment by preventing the clinical problem of PCX toxicity. As a result of these studies, it is thought that especially blank CD nanoparticles are worth researching and developing for use in cancer treatment.

## Figures and Tables

**Table 1:** Physicochemical properties of blank or paclitaxel-loaded amphiphilic cyclodextrin nanoparticles (n = 3, ±SD) [31,33].

Formulation	Particle Size (nm) ±SD	Zeta Potential (mV) ±SD	Encapsulation Efficacy (%) ± SD
Blank 6OCaproβCD nanoparticle	104 ± 1.1	−24 ± 0.3	-
PCX-loaded 6OCaproβCD nanoparticle	113 ± 4.0	−29 ± 2.0	41 ± 2.3
Blank PC βCDCD nanoparticle	75 ± 2.1	+61 ± 1.4	-
PCX-loaded PC βCDCD nanoparticle	82 ± 2.0	+62 ± 1.1	64 ± 1.9

## Data Availability

Data is contained within the article. Raw data of the mothodology is also available upon request.
